# Demographic and contextual factors as moderators of the relationship between pet ownership and health

**DOI:** 10.1080/21642850.2021.1963254

**Published:** 2021-08-06

**Authors:** Megan K. Mueller, Erin K. King, Kristina Callina, Seana Dowling-Guyer, Emily McCobb

**Affiliations:** a Department of Clinical Sciences & Center for Animals and Public Policy, Cummings School of Veterinary Medicine at Tufts University; b Tisch College of Civic Life at Tufts University; c Lynch Research Associates

**Keywords:** Pet ownership, sociodemographics, psychosocial health

## Abstract

Objective: Companion animals are an important feature of the family system, and human-animal interaction is increasingly being recognized as an important social determinant of health. However, there is a need for more nuanced assessment of not only who owns pets, but how pet ownership is related to various health outcomes, and which sociodemographic and contextual factors moderate these associations.

Design: Secondary analysis of cross-sectional data collected from an online, probability-based panel to generate a nationally representative sample of adults in the United States (*n* = 1267). Data were analyzed using hierarchical and logistic regression models.

Results: Results suggested that pet owners are systematically different from non-pet owners on a number of key demographic and contextual characteristics, and these differences vary somewhat when looking at dog owners and cat owners. When controlling for individual and family-level covariates, pet ownership was not associated with overall health status or Body Mass Index, but dog ownership was associated with higher levels of physical activity. Pet ownership was associated with higher odds of having an anxiety disorder with gender moderating this relationship, but this association was not present for dog or cat owners, suggesting this relationship is limited to other types of pets. Higher odds of depression were associated with pet ownership (for both dog and cat owners), with employment status significantly moderating the relationship between dog ownership status and depression.

Conclusions: These findings suggest that pet ownership is a complex and context specific phenomenon. Future research should carefully consider and control for sociodemographic predictors and use measurement and analysis approaches sensitive to the variations in human-animal relationships to fully understand how pets contribute to individual and family health and well-being.

## Introduction

Pet ownership is a common feature of the family context in the United Sates, with recent estimates indicating that the majority of families have at least one pet in the home (APPA, 2021). Given the high prevalence of pet ownership, there has been a corresponding interest in research assessing how companion animals may contribute to quality of life and well-being. Existing research does find some positive associations between human-animal interaction (HAI) and both physical and mental health outcomes. A number of studies have demonstrated that pet ownership is associated with reduced cardiovascular responses to stress (e.g. Allen, Shykoff, & Izzo, 2001; Friedmann, Thomas, Son, Chapa, & McCune, 2013), with one recent systematic review finding lower cardiovascular mortality rates for dog owners (Kramer, Mehmood, & Suen, 2019). One hypothesized pathway for this effect has been increased physical activity level, with some research showing higher levels of activity level and exercise in dog owners compared to non-dog owners (Mein & Grant, 2018). With regard to mental health outcomes, there is some initial evidence that pet owners are less lonely than non-pet owners (Stanley, Conwell, Bowen, & Van Orden, 2014), and another recent study demonstrated that the presence of an animal can positively impact affect (Janssens et al., 2020). Some of these effects may even be related to childhood exposure; Yolken et al. (2019) found that exposure to pets in childhood was associated with decreased risk of later mental health diagnoses (schizophrenia and bipolar disorder).

Despite some of these positive findings, there is also a substantial body of research that has demonstrated mixed, null, or negative findings. Westgarth et al. (2012) found no evidence that childhood obesity was influenced by dog ownership, and more recently, Powell et al. (2020) reported mixed findings for the relationship between oxytocin and heart rate variability responses to dog walking. The association between pet ownership and mental health outcomes has been particularly mixed regarding depression, with multiple studies finding higher rates of depression and/or depressive symptoms in pet owners as compared to non-pet owners (Bradley & Bennett, 2015; Mueller, Gee, & Bures, 2018; Sharpley et al., 2020).

The variability in findings points to the complexity of human-animal relationships and the difficulty of assessing and adequately controlling for confounding variables that can impact the degree to which pet ownership predicts physical and mental health and well-being. Pets are part of a dynamic family system that is shaped by numerous individual and contextual factors, and these factors may contribute to how pet ownership relates to well-being. Factors such as cultural/ethnic identity, gender identity, marital and family status, socioeconomic status, and housing type all contribute to the family system, but are not consistently controlled for in studies on pet ownership (Kazdin, 2015; Purewal et al., 2017; Rodriguez, Herzog, & Gee, 2021). In fact, several studies have found that health effects associated with pet ownership were attenuated or changed when controlling for confounding variables (Goh et al., 2020; Jacobson & Chang, 2018; Marsa-Sambola et al., 2016; Miles, Parast, Babey, Griffin, & Saunders, 2017) or assessing interactions between pet ownership and demographic variables (Liu et al., 2019; Stanley et al., 2014). To inform evidence-based demographic covariates for pet ownership studies, several studies in the United Kingdom have specifically assessed the sociodemographics of pet ownership (e.g. Marsa-Sambola et al., 2016; Westgarth et al., 2010, 2012). Similarly, there has been a recent increase in the inclusion of pet ownership items in large, population representative surveys in the United States (Bures, 2021). However, there is still a need for understanding which sociodemographic variables have the most predictive value for pet ownership in samples from the United States, to better inform future research design.

Because random assignment to pet ownership is typically not feasible or even ethical, identifying systematic and potentially a priori differences between pet owners and non-pet owners is important in understanding the relationship between companion animal ownership and health outcomes. Identifying these systematic differences in representative datasets may help future studies design ways to mitigate the selection effects regarding who chooses to own a pet (and what kind of pet). Furthermore, this information is needed to understand how different sociodemographic factors may mediate or moderate any outcomes associated with pet ownership. These nuances are critical in framing research that is equitable and addresses systematic inequities in healthcare and access to veterinary care. HAI research has historically struggled with representation in sampling, and understanding demographic profiles of pet ownership is an important component of accurately assessing the relationship between pets and health for diverse communities. However, it is still unclear which demographic and contextual variables have the most predictive value in modeling the health effects of pet ownership.

Furthermore, there is evidence of differential effects between different species of pets, particularly with regard to dog and cat owners. Dog ownership has been associated more strongly with positive physical health outcomes (Carr, Taylor, Gee, & Sachs-Ericsson, 2020; Friedmann et al., 2013; Gadomski, Scribani, Krupa, & Jenkins, 2017; Mein & Grant, 2018; Westgarth et al., 2019), perhaps due to dog walking or other features of the relationship related to physical activity (Antonacopoulos & Pychyl, 2017). Some initial evidence has also suggested that dog ownership may be related to more positive mental health outcomes compared to cat ownership (Bao & Schreer, 2016), and that cat owner personality interacts in complex ways with cat behavior and welfare parameters (Finka, Ward, Farnworth, & Mills, 2019). Therefore, it is important to assess these potential differential relationships to health outcomes by species in the context of demographic and contextual confounders.

The existing research points to a need for more nuanced assessment of who owns pets, and how the sociodemographic and contextual factors associated with having a companion animal may moderate any relationships between pet ownership and health outcomes (Rodriguez et al., 2021). To address this gap, the purpose of this study was to explore the nature of these systematic differences using a representative sample of adults in the United States to assess the following research questions:

1. *Who owns pets?* We examined differences in individual and contextual-level demographics between pet owners and non-pet owners, and whether these differences varied by dog ownership and cat ownership.
2. *Are pets associated with indicators of health?* We examined whether pet ownership is associated with indicators of physical and mental health, and whether these associations are moderated by sociodemographic variables.

The goal of this research is to inform future population-representative studies of pet ownership as a social determinant of health, and to provide suggestions for empirically identified covariates and potential moderators that should be incorporated into analyses.

## Method

### Participants

Participants for this study (*n *= 1,267) were recruited using the Ipsos market research company. Data were collected as part of a larger study conducted by the Tufts University Research Group on Equity in Health, Wealth, and Civic Engagement through the KnowledgePanel, an online, probability-based panel that is representative of the United States population. Eligible participants included adults ages 18 years and older who were non-institutionalized and living in the United States. Demographics of the sample are reported in the results.

### Procedure

Participants were initially recruited by Ipsos via random sampling of telephone numbers and residential addresses, and then invited by telephone or mail to participate. Participants who did not have access to the internet were provided with a laptop and internet connection at no cost by Ipsos. Participants received a unique log-in to complete online surveys and were sent emails several times per month to participate in research.

A representative sample of 1,980 participants were invited to complete the survey, which was fielded in English and Spanish and available from May 29 to June 10, 2020. All non-responding sample individuals were sent an automatic reminder email on day three, and additional reminders were sent to Black and Hispanic members on day 11 for purposes of oversampling. Median completion time for the survey was 17 minutes, and participants received a standard incentive payment (typically equivalent of 1,000 points, or $1), as well as an entry into a sweepstakes through KnowledgePanel. Of the 1,980 panel members, 1,267 were eligible and responded, with a final completion rate of 64%. For further information on the sampling frame and the Equity in Health, Wealth, and Civic Engagement Survey, see Stopka et al. (2021) and https://equityresearch.tufts.edu/the-data/. All research was approved by the Tufts University Institutional Review Board.

### Measures

#### Demographic/Contextual variables

Participants were asked standard demographic questions such as age (continuous variable), gender identity (male, female), and racial/ethnic identity (White/non-Hispanic, Black/non-Hispanic, Hispanic, other/non-Hispanic, 2+ races/Non-Hispanic). Due to small sample sizes, 2+ races/non-Hispanic and other/non-Hispanic were combined into one category. Participants were asked to select their highest level of education and could choose less than high school, high school, some college, or bachelor's degree or higher. Income status was measured incrementally in 21 categories starting with ‘less than $5,000’ (1) and ending with ‘$250,000 or more’ (21).

Participants were also asked to report marital status (married, widowed, divorced, separated, never married, living with partner). The presence of children in the house was measured by a dichotomous yes/no variable that was derived from a series of continuous variables in which participants reported the number of members in their household younger than 1, between the ages of 2 and 5, between the ages of 6 and 12, and between the ages of 13 to 17.

Employment status was recoded into a dichotomous variable (working or not working) from a list of employment options (working as a paid employee, working-self-employed, not working-on temporary layoff from a job, not working-looking for work, not working–retired, not working-disabled, not working-other). Participants were also asked to select their housing type: a one-family house detached, a one-family house attached, a building with 2+ apartments, a mobile home, or a boat, RV, van, etc. Due to small sample sizes in the categories of mobile home, boat, RV, and van, these categories were combined. Urbanicity of housing environment was measured through self-reported options and participants could select urban, suburban or rural.

#### Pet ownership

Pet ownership was measured by asking participants if they owned a pet, and if yes, what species they owned (multiple-select variable for dog, cat, fish, bird, gerbil, reptile, horse, other). Pet ownership was then categorized into three dichotomous variables: pet ownership (pet owners vs. non-pet owners), dog ownership (dog owners vs. non-dog owners, including both non-pet owners and owners of non-dog pets), and cat ownership (cat owners vs. non-cat owners, including both non-pet owners and owners of non-cat pets). These dichotomous categorizations are not mutually exclusive, but instead intended to assess the unique effects of owning a specific species of animal.

#### Health indicators

Participants answered a series of questions related to indicators of health and well-being. General physical health was measured by a single select, self-reported item in which participants were asked to rate their physical health as 1 = excellent, 2 = very good, 3 = good, 4 = fair, or 5 = poor. Body Mass Index (BMI) was derived from self-reported height and weight. Participants also reported how often they participate in light to moderate physical activities for at least ten minutes a day (defined as activities that cause light sweating or a slight to moderate increase in breathing or heart rate; 1 = never, 2 = less than once per week, 3 = 1-2 times per week, 4 = 3-5 times per week, 5 = 6 or more times a week).

As an indicator of physical disability status, participants were asked if they or anyone in their household had a long-lasting condition that substantially limited one or more basic physical activities (response options included: no, yes-myself only, yes-someone else only, or yes-both myself and someone else). This measure was recoded into a dichotomous variable to represent whether the reporting participant self-identified as having a physical disability. Similarly, participants were also asked if they or someone in their household had a long-lasting physical, mental or emotional condition that increases the difficulty of learning, remembering or concentrating (referred to in this analysis as ‘cognitive disability’; response options included no, yes-myself only, yes-someone else only, or yes-both myself and someone else). This variable was also recoded to a dichotomous variable reflecting whether the participant self-identified as having a cognitive disability. Finally, participants reported if they had been diagnosed with an anxiety disorder and/or depression (both dichotomous yes/no variables).

### Data analysis

The sample was weighted to match the United States’ population with regard to gender, age, race, ethnicity, education, Census region, income, owning a home, and urbanicity. Sampling weights were based on the 2019 Current Population supplement and ranged from 0.174 to 3.7 with a median of 0.843. Additional details reported in Stopka et al. (2021).

All analyses were performed in SPSS version 27.0. Weighted descriptive statistics and frequencies are reported for all pet ownership, demographic, and health indicator variables. The first research question (*Who owns pets?*) was assessed with binomial logistic regression models with the continuous and categorical sociodemographic predictors of education level, gender, housing type, urbanicity, income, marital status, presence of children in the home, employment status, and race/ethnicity as predictors of pet ownership, dog ownership, and cat ownership. The second research question (*Are pets associated with indicators of health?*) was tested using a series of hierarchical regression models for each health indicator outcome. We used multiple regression for continuous outcomes (self-reported health status, BMI, light/moderate physical activity), and binomial logistic regression for dichotomous outcomes (physical disability, cognitive disability, anxiety disorder, depression).

Variables were tested for the necessary assumptions for regression modeling, including independence of observations, linearity, homoscedasticity, normal distribution of residuals, and multicollinearity (see Supplementary Materials for additional information). To interpret significance, we report *p* values, 95% confidence intervals, and effect sizes to support interpretation of the significance and meaningfulness of our findings. Prior to analysis, power analyses were conducted in G*Power 3.1.9.4.31. For analyses for up to 15 predictors, the sample provides the power of .80 to detect effect sizes as small as 0.006. For logistic regression models, model fit was assessed using the chi-square test of model coefficients and Nagelkerke R^2^. For hierarchical multiple regression models, model fit was assessed using R^2^ values, *p* <.05.

For each outcome, Model 1 includes pet ownership as a predictor, Model 2 includes pet ownership and the significant demographic/contextual predictors identified in the first research question. Where pet ownership and demographic variables were significant predictors of health indicators in Model 2, we examined potential interaction effects in Model 3. These hierarchical models were repeated for dog ownership and cat ownership with significant demographic/contextual predictors identified in the first research question.

## Results

Respondents ranged in age from 18 to 94 years with an average age of 48 years (*SD* = 17.57); there were 613 (48.4%) participants who identified as male, and 654 (51.6%) who identified as female. For self-reported racial/ethnic identity, 800 (63.1%) identified as White/non-Hispanic, 150 (11.8%) identified as Black/non-Hispanic, 208 (16.4%) identified as Hispanic, and 109 (8.6%) identified as other/2+ races, Non-Hispanic. Education level of participants varied with 134 (10.6%) completing less than high school, 359 (28.3%) completing high school, 352 (27.8%) completing some college, and 422 (33.3%) completing a bachelor’s degree or higher.

Of the 1,267 participants, 750 (59.2%) were pet owners, and 517 (40.8%) not owning any pets. Of the 750 pet owners, 504 (67.2%) owned dogs (290 owned only a dog and no other pets), 356 (47.4%) owned cats (167 owned only a cat and no other pets), and 179 (23.9%) owned other types of pets. On average pet owners reported having an average of 1.45 animals in their home (range 1-8, *SD = *.76). Demographic and contextual variables stratified by pet ownership status are presented in Table 1.

**Table 1.** Weighted descriptive statistics/frequencies for demographic/contextual and health indicators, stratified by pet ownership status.

	Pet Owners	Non-Pet Owners	Dog Owners	Non-Dog Owners	Cat Owners	Non-Cat Owners
**Demographic/Contextual Indicators**	*n* = 750	*n* = 517	*n* = 504	*n* = 763	*n* = 356	*n* = 911
	*M(SD)*	*M(SD)*	*M(SD)*	*M(SD)*	*M(SD)*	*M(SD)*
Household Income*	13.27 (4.79)	13.10 (4.57)	13.51 (4.66)	12.99 (4.71)	13.03 (4.96)	13.26 (4.59)
Gender	*n (%)*	*n (%)*	*n (%)*	*n (%)*	*n (%)*	*n (%)*
Female	407 (54.3%)	247 (47.8%)	277 (55%)	377 (49.4%)	188 (52.8%)	466 (51.1%)
Male	343 (45.7%)	270 (52.2%)	227 (45%)	386 (50.6%)	168 (47.2%)	446 (48.9%)
Race/Ethnicity						
White, Non-Hispanic	514 (68.5%)	286 (55.2%)	342 (67.9%)	458 (60.0%)	271 (76.1%)	529 (58.1%)
Black, Non-Hispanic	59 (7.9%)	91 (17.6%)	36 (7.1%)	114 (14.9%)	25 (7.0%)	124 (13.6%)
Other/Multi-racial, Non-Hispanic	53 (7.1%)	56 (10.8%)	36 (7.1%)	73 (9.6%)	23 (6.5%)	86 (9.5%)
Hispanic	124 (16.5%)	85 (16.4%)	90 (17.9%)	118 (15.5%)	37 (10.4%)	171 (18.8%)
Education						
Less than High School	91 (12.1%)	44 (8.5%)	59 (11.7%)	75 (9.8%)	37 (10.4%)	97 (10.6%)
High School	215 (28.7%)	144 (27.8%)	152 (30.2%)	206 (27.0%)	112 (31.5%)	247 (27.1%)
Some College	223 (29.7%)	129 (24.9%)	147 (29.2%)	205 (26.9%)	111 (31.3%)	240 (26.3%)
Bachelor's degree +	221 (29.5%)	201 (38.8%)	145 (28.8%)	277 (36.3%)	95 (26.8%)	327 (35.9%)
Housing Type						
One family, detached	563 (75.1%)	334 (64.6%)	414 (82.1%)	484 (63.4%)	248 (69.9%)	649 (71.2%)
One family, attached	54 (7.2%)	52 (10.1%)	32 (6.3%)	74 (9.7%)	27 (7.6%)	79 (8.7%)
Multi-family building	96 (12.8%)	122 (23.6%)	30 (6.0%)	188 (24.6%)	60 (16.9%)	158 (17.3%)
Mobile home, boat, car	37 (4.9%)	9 (1.7%)	28 (5.6%)	18 (2.4%)	20 (5.6%)	25 (2.7%)
Urbanicity						
Urban	253 (33.8%)	200 (38.7%)	158 (31.4%)	295 (38.7%)	117 (32.9%)	336 (36.9%)
Rural	168 (22.5%)	53 (10.3%)	133 (26.4%)	88 (11.5%)	90 (25.3%)	131 (14.4%)
Suburban	327 (43.7%)	264 (51.1%)	212 (42.1%)	380 (49.8%)	149 (41.9%)	443 (48.7%
Marital Status						
Married	408 (54.4%)	266 (51.5%)	287 (56.9%)	387 (50.8%)	190 (53.2%)	485 (53.2%)
Widowed	29 (3.9%)	31 (6.0%)	24 (4.8%)	36 (4.7%)	14 (3.9%)	47 (5.2%)
Divorced	71 (9.5%)	49 (9.5%)	45 (8.9%)	75 (9.8%)	35 (9.8%)	85 (9.3%)
Separated	14 (1.9%)	7 (1.4%)	8 (1.6%)	13 (1.7%)	9 (2.5%)	12 (1.3%)
Never married	167 (22.3%)	141 (27.3%)	115 (22.8%)	193 (25.3%)	80 (22.4%)	229 (25.1%)
Living with partner	61 (8.1%)	23 (4.4%)	25 (5.0%)	58 (7.6%)	29 (8.1%)	54 (5.9%)
Children						
Children	212 (28.3%)	111 (21.5%)	159 (31.5%)	165 (21.6%)	91 (25.6%)	233 (25.6%)
No Children	538 (71.7%)	406 (78.5%)	345 (68.5%)	598 (78.4%)	265 (74.4%)	678 (74.4%)
Employment						
Employed	532 (71%)	294 (56.8%)	361 (71.5%)	465 (60.9%)	247 (69.4%)	579 (63.6%)
Unemployed	217 (29%)	224 (43.2%)	144 (28.5%)	298 (39.1%)	109 (30.6%)	332 (30.6%)
						
**Health Indicators**	*M(SD)*	*M(SD)*	*M(SD)*	*M(SD)*	*M(SD)*	*M(SD)*
Health Status	2.60 (0.98)	2.54 (0.99)	2.58 (0.99)	2.58 (0.99)	2.66 (1.00)	2.55 (0.98)
Body Mass Index	28.48 (6.43)	28.03 (6.45)	28.61 (6.30)	28.09 (6.53)	28.53 (6.52)	28.20 (6.41)
Physical activity	3.26 (1.22)	3.17 (1.20)	3.35 (1.21)	3.14 (1.21)	3.20 (1.25)	3.23 (1.20)
	*n (%)*	*n (%)*	*n (%)*	*n (%)*	*n (%)*	*n (%)*
Physical Disability	94 (12.8%)	63 (12.5%)	62 (12.5%)	95 (12.7%)	51 (14.6%)	105 (11.8%)
Cognitive Disability	65 (8.8%)	25 (5.0%)	37 (7.5%)	53 (7.1%)	40 (11.4%)	50 (5.6%)
Anxiety Disorder	92 (12.7%)	44 (8.8%)	58 (11.8%)	79 (10.8%)	47 (13.6%)	89 (10.1%)
Depression	132 (18.1%)	49 (9.8%)	83 (16.9%)	98 (13.4%)	66 (19.1%)	115 (13.1%)

*Note: Income measured as 21 discrete categories treated as continuous data; an income level of 13 corresponds to $60,000-$75,000 USD.

### Demographic and contextual predictors of pet ownership

The logistic regression models assessing demographic and contextual predictors of pet ownership were statistically significant, for overall pet ownership (χ^2^(20) = 164.36, *p* < .001; Nagelkerke R^2 ^= 0.164), dog ownership (χ^2^(20) = 182.59, *p* < .001; Nagelkerke R^2 ^= 0.182), and cat ownership (χ^2^(20) = 81.65, *p* < .001; Nagelkerke R^2 ^= 0.09). Results showed that income and marital status were not significant predictors of pet ownership, dog ownership, or cat ownership. Gender significantly predicted overall pet ownership, with female participants more likely to own pets than male participants, but there were no gender differences by dog or cat ownership status. Racial/ethnic identity was also a significant predictor of all types of pet ownership with Black/non-Hispanic, Hispanic, and Multiracial/other/non-Hispanic participants less likely to own pets overall than White/non-Hispanic participants. Looking at cat and dog ownership, Black/non-Hispanic, Hispanic, and Multiracial/other/non-Hispanic participants were less likely to own cats than White/non-Hispanic participants and Black/non-Hispanic participants were less likely to own dogs than White/non-Hispanic individuals, although there were no other significant differences for dog ownership by race/ethnicity.

Individuals with a bachelor’s degree education or higher were less likely to own a pet than those with less than a high school education, with the same patterns for dog and cat ownership. With regard to housing, participants living in multi-family buildings were less likely to own a pet overall, as well as less likely to own a dog compared to those who lived in one-family, detached housing. Cat ownership was not predicted by housing type. Participants who lived in rural settings were more likely to own a pet than those in urban settings (including both dogs and cats), and those in suburban settings were also less likely to own a dog as compared to those living in an urban setting.

Having children in the home was also associated with higher odds of overall pet ownership as well as dog ownership, but not cat ownership. Participants who were employed were more likely to own pets (including both cats and dogs) compared to those who were not employed. See Table 2 for full regression results, including odds ratios and confidence intervals.

**Table 2.** Weighted logistic regression results for demographic/contextual variables predicting pet ownership, dog ownership, and cat ownership.

	**Pet Ownership**						**Dog Ownership**						**Cat Ownership**				
	B	S.E.	*p*	OR	95% C.I. for OR		B	S.E.	*p*	OR	95% C.I. for OR		B	S.E.	*p*	OR	95% C.I. for OR
Household Income	<.001	0.02	0.97	1.00	0.97-1.03		0.01	0.02	0.53	1.01	0.98-1.04		0.01	0.02	0.78	1.01	0.97-1.04
Gender																	
Female*																	
Male	0.30	0.13	0.02	1.35	1.05-1.72		0.23	0.13	0.08	1.25	0.98-1.61		0.07	0.13	0.58	1.08	0.83-1.40
Race/Ethnicity																	
White, Non-Hispanic*			<.001						0.016						<.001		
Black, Non-Hispanic	-1.00	0.20	<.001	0.37	0.25-0.55		-0.71	0.23	0.00	0.49	0.32-0.77		-0.95	0.25	<.001	0.39	0.24-0.62
Other/Multi-racial, Non-Hispanic	-0.50	0.22	0.02	0.61	0.40-0.93		-0.17	0.23	0.47	0.84	0.54-1.33		-0.58	0.25	0.02	0.56	0.34-0.93
Hispanic	-0.46	0.18	0.01	0.63	0.44-0.91		-0.03	0.19	0.89	0.98	0.68-1.40		-1.03	0.22	<.001	0.36	0.24-0.55
Education																	
Less than high school*			<.001						0.04						0.002		
High School	-0.44	0.24	0.06	0.64	0.40-1.02		-0.05	0.23	0.82	0.95	0.61-1.49		-0.12	0.24	0.63	0.89	0.55-1.43
Some College	-0.35	0.24	0.14	0.70	0.44-1.13		-0.14	0.23	0.56	0.87	0.55-1.38		-0.08	0.25	0.76	0.93	0.57-1.50
Bachelor's degree +	-0.93	0.25	<.001	0.39	0.24-0.64		-0.50	0.24	0.04	0.61	0.38-0.98		-0.69	0.26	0.01	0.50	0.30-0.84
Housing Type																	
One family detached*			<.001						<.001						0.27		
One family attached	-0.21	0.22	0.34	0.81	0.52-1.25		-0.43	0.24	0.07	0.65	0.41-1.04		0.21	0.25	0.39	1.24	0.76-2.02
Multi-family building	-0.77	0.18	<.001	0.46	0.33-0.66		-1.59	0.22	<.001	0.20	0.13-0.32		0.17	0.20	0.38	1.19	0.81-1.74
Mobile home	0.89	0.41	0.03	2.43	1.08-5.45		0.58	0.34	0.08	1.79	0.93-3.47		0.58	0.33	0.08	1.79	0.94-3.40
Urbanicity																	
Urban*			0.001						<.001						0.02		
Rural	0.45	0.21	0.03	1.56	1.04-2.35		0.49	0.19	0.01	1.64	1.12-2.39		0.39	0.20	0.048	1.48	1.00-2.17
Suburban	-0.25	0.14	0.08	0.78	0.59-1.03		-0.29	0.15	0.04	0.75	0.56-0.99		-0.12	0.16	0.42	0.88	0.65-1.20
Marital Status																	
Married*			0.064						0.78						0.31		
Widowed	-0.37	0.30	0.22	0.69	0.39-1.24		0.16	0.30	0.60	1.17	0.65-2.13		-0.37	0.34	0.27	0.69	0.36-1.34
Divorced	0.05	0.23	0.83	1.05	0.67-1.65		0.05	0.24	0.82	1.05	0.67-1.67		-0.02	0.24	0.94	0.98	0.61-1.58
Separated	0.37	0.50	0.45	1.45	0.55-3.84		0.01	0.50	0.98	1.01	0.38-2.69		0.84	0.49	0.09	2.31	0.89-5.99
Never married	-0.03	0.16	0.85	0.97	0.71-1.33		0.14	0.16	0.39	1.15	0.84-1.59		-0.06	0.17	0.73	0.94	0.67-1.32
Living with partner	0.77	0.29	0.01	2.16	1.23-3.77		-0.29	0.28	0.31	0.75	0.43-1.30		0.27	0.26	0.30	1.31	0.78-2.20
Children																	
No Children*																	
Children	0.31	0.15	0.04	1.36	1.02-1.82		0.49	0.15	<.001	1.63	1.23-2.17		0.06	0.15	0.72	1.06	0.78-1.43
Employment																	
Unemployed*																	
Employed	0.85	0.14	<.001	2.34	1.77-3.10		0.66	0.15	<.001	1.94	1.45-2.58		0.41	0.15	0.01	1.50	1.11-2.03

Note: *indicates reference group category; OR = odds ratio

### Pet ownership and self-reported health status

In the overall sample, average self-reported health status was 2.58 (*SD = *0.99, range 1 [excellent] to 5 [poor]). Health status stratified by pet ownership is reported in Table 1. Model 1 with pet ownership alone predicting self-reported health status was not statistically significant; R^2^ = .001, *F*(1, 1250) = 1.06, *p* = .30, adjusted R^2^ = <.001. Model 2, which included pet ownership and demographic/contextual covariates was overall statistically significant (R^2^ = .09, *F*(15, 1236) = 8.50, *p* <.001, adjusted R^2^ = .082), but pet ownership was not significantly associated with health status.

Similarly, for dog ownership, Model 1 with dog ownership alone predicting self-reported health status was not significant; R^2^ <.001, *F*(1, 1250) = .013, *p* = .91, adjusted R^2^ = <.001. Model 2 was statistically significant (R^2^ = .09, *F*(14, 1237) = 7.98, *p* <.001, adjusted R^2^ = .08), with dog ownership not associated with health status. Cat ownership as a predictor of health status demonstrated similar results, with Model 1 not significantly predicting health status; R^2^ = .003, *F*(1, 1250) = 3.80, *p* = .06, adjusted R^2^ = .002. Model 2 was statistically significant (R^2^ = .08, *F*(10, 1241) = 11.44, *p* <.001, adjusted R^2^ = .08), but cat ownership was not significantly related to health status. See Table 3 for pet, dog, and cat ownership parameters, full regression parameters for all covariates in all models are reported in Supplemental Tables 1, 2 and 3.

**Table 3.** Weighted hierarchical regression models for pet ownership, dog ownership, and cat ownership predicting continuous health indicators.

	B	SE	β	*t*	*p*	95% CI	f2
**Health Status**							
Model 1 - Pet Ownership, no covariates	0.06	0.06	0.03	1.03	0.30	-0.05-0.17	0.001
Model 2 - Pet Ownership with covariates	0.09	0.06	0.04	1.49	0.14	-0.03-0.20	0.002
Model 1 - Dog Ownership, no covariates	-0.01	0.06	0.00	-0.11	0.91	-0.12-0.11	0.000
Model 2 - Dog Ownership with covariates	0.04	0.06	0.02	0.73	0.47	-0.07-0.16	0.000
Model 1 - Cat Ownership, no covariates	0.12	0.06	0.05	1.90	0.06	0.00-0.24	0.003
Model 2 - Cat Ownership with covariates	0.10	0.06	0.05	1.60	0.11	-0.02-0.22	0.002
**BMI**							
Model 1 - Pet Ownership, no covariates	0.46	0.38	0.04	1.22	0.22	-0.28-1.21	0.001
Model 2 - Pet Ownership with covariates	0.32	0.39	0.02	0.81	0.42	-0.46-1.09	0.001
Model 1 - Dog Ownership, no covariates	0.54	0.38	0.04	1.43	0.15	-0.20-1.29	0.002
Model 2 - Dog Ownership with covariates	0.39	0.40	0.03	0.98	0.33	-0.39-1.17	0.001
Model 1 - Cat Ownership, no covariates	0.32	0.41	0.02	0.78	0.44	-0.49-1.12	0.000
Model 2 - Cat Ownership with covariates	0.20	0.41	0.01	0.48	0.63	-0.61-1.01	0.000
**Physical activity**							
Model 1 - Pet Ownership, no covariates	0.09	0.07	0.04	1.26	0.21	-0.05-0.23	0.001
Model 2 - Pet Ownership with covariates	0.02	0.07	0.01	0.28	0.78	-0.12-0.16	0.000
Model 1 - Dog Ownership, no covariates	0.21	0.07	0.09	3.03	&lt;0.001	0.07-0.35	0.007
Model 2 - Dog Ownership with covariates	0.16	0.07	0.07	2.28	0.02	0.02-0.30	0.004
Model 1 - Cat Ownership, no covariates	-0.03	0.08	-0.01	-0.45	0.66	-0.18-0.12	0.000
Model 2 - Cat Ownership with covariates	-0.07	0.08	-0.03	-0.94	0.35	-0.22-0.08	0.001

Note: Model 1 includes pet/dog/cat ownership as single predictors, Model 2 includes covariates. Full regression parameters for all covariates are included in the supplementary materials.

### Pet ownership and Body Mass Index

Body Mass Index (BMI) ranged from 16.14 to 53.21 in the overall sample (*M *= 28.30; *SD* = 6.44). BMI stratified by pet ownership is reported in Table 1. Model 1 with pet ownership alone predicting BMI was not statistically significant R^2^ = .001, *F*(1, 1199) = 1.91, *p* = .22, adjusted R^2^ < .001. Model 2 with pet ownership and demographic/contextual covariates did significantly predict BMI (R^2^ = .06, *F*(15, 1185) = 5.01, *p* <.001, adjusted R^2^ = .05), but pet ownership was not associated with BMI in either model.

Dog ownership alone also did not significantly predict BMI (Model 1: R^2^ = .002, *F*(1, 1199) = 2.05, *p* = .15, adjusted R^2^ = .001). When demographic/contextual covariates were included, Model 2 significantly predicted BMI (R^2^ = .06, *F*(14, 1188) = 5.16, *p* <.001, adjusted R^2^ = .05), but dog ownership was not a significant predictor. Similarly, Model 1 with cat ownership as a predictor of BMI was not significant (R^2^ = .001, *F*(1, 1199) = 0.60, *p* = .44, adjusted R^2^ <.001), and Model 2 with covariates significantly predicted BMI but cat ownership was not associated with BMI (R^2^ = .06, *F*(10, 1190) = 6.87, *p* <.001, adjusted R^2^ = .05). See Table 3 for pet, dog, and cat ownership parameters, full regression parameters for all covariates in all models are reported in Supplemental Tables 4, 5, and 6.

### Pet ownership and light/moderate physical activity

In the whole sample, frequency of light/moderate physical activity for 10 or more minutes ranged from 1 (never) to 5 (6 or more times a week), with an average of 3.22 (*SD* = 1.21). Model 1 with pet ownership as the sole predictor did not significantly predict physical activity (R^2^ = .001, *F*(1, 1246) = 1.58, *p* = .21, adjusted R^2^ <.001). Model 2 with pet ownership and demographic/pet ownership covariates did significantly predict physical activity (R^2^ = .06, *F*(15, 1232) = 8.63, *p* <.001, adjusted R^2^ = .08), but pet ownership was not associated with activity level.

For dog ownership, Model 1 with dog ownership as the sole predictor did significantly predict physical activity (R^2^ = .007, *F*(1, 1246) = 9.15, *p* = .003, adjusted R^2^ = .006), with dog ownership positively associated with higher levels of activity (see Table 3). Model 2 with demographic/covariates was also statistically significant (R^2^ = .10, *F*(14, 1233) = 12.91, *p* <.001, adjusted R^2^ = .09), and dog ownership remained significantly associated with higher levels of activity. Model 3 assessed the presence of any interaction effects between dog ownership and significant covariates (identifying as Black/non-Hispanic, having a high school education, having some college education, having a bachelor’s degree or higher, and being employed). No significant interaction effects were identified (see Supplemental Table 8 for parameters), and Model 3 did not predict significantly more of the variance than Model 2 (R^2^ change = .003, *F*(5, 1227) = 2.10, *p* = .06). Therefore, Model 2 is the most parsimonious model for predicting physical activity level.

Model 1 with cat ownership predicting physical activity was not statistically significant, R^2^ <.001, *F*(1, 1246) = 0.20, *p* = .66, adjusted R^2^ <.001. Model 2 with demographic/covariate predictors was statistically significant (R^2^ = .09, *F*(10, 1237) = 12.31, *p* <.001, adjusted R^2^ = .08), but cat ownership was not associated with physical activity level. See Table 3 for pet, dog, and cat ownership parameters, full regression parameters for all covariates and interaction terms in all models are reported in Supplemental Tables 7, 8, and 9.

### Pet ownership and disability status

Overall, 157 (12.4%) of participants reported having a long-term physical disability, and 90 (7.1%) had a long-term cognitive-related disability (see Table 1 for disability status stratified by pet ownership). For pet ownership, Model 1 was not statistically significant in predicting physical disability status, χ^2^(1) = 0.02, *p* = .88, Nagelkerke R^2^ <.001. Model 2 with demographic/contextual covariates did significantly predict physical disability status, χ^2^(15) = 111.98, *p* <.001, Nagelkerke R^2^ = .16, but pet ownership status did not predict the odds of having a physical disability (see Table 4). Similar patterns emerged for dog ownership and cat ownership models; Model 1 for dog ownership did not significantly predict physical disability status, χ^2^(1) = 0.01, *p* = .91, Nagelkerke R^2^ <.001; Model 2 was significant (χ^2^[14] = 111.54, *p* <.001, Nagelkerke R^2^ = .16) but dog ownership was not associated with odds of having a physical disability. Cat ownership similarly did not predict physical disability status in Model 1, χ^2^(1) = 1.78, *p* = .18, Nagelkerke R^2^ = .003, and was not a significant predictor within Model 2 (χ^2^[10] = 101.99, *p* <.001, Nagelkerke R^2^ = .15). Full regression parameters for all covariates and interactions in all models are reported in Supplemental Tables 10, 11, and 12.

**Table 4.** Weighted logistic regression models for pet ownership, dog ownership, and cat ownership predicting dichotomous health indicators.

	B	S.E.	Wald χ^2^	*p*	OR	95% CI
**Physical Disability**						
Model 1 - Pet Ownership, no covariates	0.03	0.17	0.02	0.88	1.03	0.73-1.44
Model 2 - Pet Ownership with covariates	0.22	0.20	1.19	0.28	1.24	0.84-1.82
Model 1 - Dog Ownership, no covariates	-0.02	0.18	0.01	0.91	0.98	0.70-1.38
Model 2 - Dog Ownership with covariates	0.18	0.20	0.81	0.37	1.20	0.81-1.78
Model 1 - Cat Ownership, no covariates	0.25	0.18	1.82	0.18	1.28	0.89-1.84
Model 2 - Cat Ownership with covariates	0.25	0.20	1.60	0.21	1.29	0.87-1.90
**Cognitive Disability**						
Model 1 - Pet Ownership, no covariates	0.62	0.24	6.40	0.01	1.85	1.15-2.98
Model 2 - Pet Ownership with covariates	0.76	0.27	7.91	0.01	2.13	1.26-3.62
Model 1 - Dog Ownership, no covariates	0.06	0.22	0.06	0.80	1.06	0.68-1.64
Model 2 - Dog Ownership with covariates	0.32	0.26	1.58	0.21	1.38	0.84-2.27
Model 1 - Cat Ownership, no covariates	0.75	0.22	11.47	&lt;0.001	2.13	1.37-3.29
Model 2 - Cat Ownership with covariates	0.70	0.24	8.90	0.003	2.02	1.27-3.21
**Anxiety Disorder**						
Model 1 - Pet Ownership, no covariates	0.40	0.19	4.36	0.04	1.50	1.03-2.19
Model 2 - Pet Ownership with covariates	0.47	0.21	5.05	0.03	1.60	1.06-2.41
Model 1 - Dog Ownership, no covariates	0.10	0.18	0.32	0.58	1.11	0.77-1.59
Model 2 - Dog Ownership with covariates	0.36	0.20	3.07	0.08	1.43	0.96-2.13
Model 1 - Cat Ownership, no covariates	0.34	0.19	3.06	0.08	1.40	0.96-2.04
Model 2 - Cat Ownership with covariates	0.30	0.20	2.26	0.13	1.35	0.91-2.00
**Depression**						
Model 1 - Pet Ownership, no covariates	0.71	0.18	15.79	&lt;.001	2.04	1.43-2.89
Model 2 - Pet Ownership with covariates	0.80	0.19	16.83	&lt;.001	2.22	1.52-3.24
Model 1 - Dog Ownership, no covariates	0.28	0.16	2.95	0.09	1.32	0.96-1.82
Model 2 - Dog Ownership with covariates	0.50	0.18	7.59	0.01	1.65	1.15-2.34
Model 1 - Cat Ownership, no covariates	0.44	0.17	6.81	0.01	1.56	1.12-2.17
Model 2 - Cat Ownership with covariates	0.40	0.18	5.19	0.02	1.49	1.06-2.11

Note: Model 1 includes pet/dog/cat ownership as single predictors, Model 2 includes covariates. Full regression parameters for all covariates are included in the supplementary materials. OR = odds ratio

For the health indicator of cognitive-related disability, pet ownership in Model 1 was predictive of disability status, χ^2^(1) = 6.86 *p* = .009, Nagelkerke R^2^ = .14, with pet ownership being associated with higher odds of having a cognitive disability (OR: 1.85, see Table 4). In Model 2, the addition of demographic/contextual covariates strengthened model fit (χ^2^[15] = 82.12, *p* <.001, Nagelkerke R^2^ = .16), and pet ownership was associated with 2.13 higher odds of having a cognitive disability. Model 3 tested interactions between significant covariates (education level, housing type, and employment status), but no significant interactions were identified (see Supplementary Table 13) and the addition of interaction terms did not improve model fit (Model 3: χ^2^(7) = 5.34, *p* = .55).

In assessing dog ownership as a predictor of cognitive disability status, Model 1 was not significant, χ^2^(1) = .06, *p* = .80, Nagelkerke R^2^ <.001. Model 2 with covariates included was significant (χ^2^[14] = 74.7, *p* <.001, Nagelkerke R^2^ = .14), but unlike pet ownership, dog ownership was not associated with odds of having a cognitive disability (see Table 4, full parameters in Supplemental Table 14). Cat ownership in Model 1 was significantly associated with higher odds of having a cognitive disability (OR: 2.13; χ^2^[1] = 11.01, *p* = .001, Nagelkerke R^2^ = .02), and this association remained when covariates were added to Model 2 (OR: 2.02; χ^2^[10] = 58.10, *p* <.001, Nagelkerke R^2^ = .11). Model 3 then assessed interactions between significant covariates (education level and employment status), but no significant interactions were identified (see Supplementary Table 15) and the addition of interaction terms did not improve model fit (Model 3: χ^2^(4) = 6.20, *p* = .19). See Table 4 for pet, dog, and cat ownership parameters and odds ratios.

### Pet ownership and anxiety disorder

Of the overall sample, 136 participants (10.7%) reported having an anxiety disorder. Pet ownership was associated with higher odds of having an anxiety disorder (OR: 1.50; Model 1 χ^2^[1] = 4.51, *p* = .03, Nagelkerke R^2^ = .07), and this relationship was maintained when covariates were included in the model (OR: 1.60; Model 2 χ^2^[15] = 53.33, *p* <.001, Nagelkerke R^2^ = .09). Model 3 included interactions between pet ownership and significant covariates (gender, race/ethnicity, housing type), and there was a significant interaction between pet ownership and gender in predicting having an anxiety disorder (OR: 2.70, *p *= .02, 95% CI [1.20, 6.09]), suggesting that gender moderates the relationship between pet ownership and having an anxiety disorder. For female participants, having a pet was associated with higher odds (OR: 2.04) of having an anxiety disorder, while for male participants, having a pet was associated with lower odds (OR: 0.75) of having an anxiety disorder. For participants who did not have a pet, the odds of anxiety were approximately the same for females as compared to males (OR: 0.89). For those with a pet, female participants were 2.40 times more likely to have an anxiety disorder compared to male participants.

For dog ownership, Model 1 was not significant in predicting having an anxiety disorder, χ^2^(1) = .31, *p* = .58, Nagelkerke R^2^ = .001. Model 2 with covariates included was significant (χ^2^[14] = 43.29, *p* <.001, Nagelkerke R^2^ = .07), but dog ownership was not associated with the odds of having an anxiety disorder. Similarly, for cat ownership Model 1 did not predict having an anxiety disorder, χ^2^(1) = 2.98, *p* = .09, Nagelkerke R^2^ = .01. Model 2 with covariates included was significant (χ^2^[10] = 23.58, *p* = .009, Nagelkerke R^2^ = .04), but cat ownership did not predict odds of having an anxiety disorder. Table 4 includes pet, dog, and cat ownership parameters and odds ratios, full regression parameters for all covariates and interaction terms in all models are reported in Supplemental Tables 16, 17, and 18.

### Pet ownership and depression

In the overall sample, 180 individuals (14.2%) reported having depression. Pet ownership was associated with higher odds of depression (OR: 2.05; Model 1 χ^2^[1] = 16.96, *p* <.001, Nagelkerke R^2^ = .07). This association between pet ownership and depression remained when covariates were included in the model (OR: 2.22; Model 2 χ^2^[15] = 79.35, *p* <.001, Nagelkerke R^2^ = .11). Model 3 tested for interaction effects between pet ownership and significant covariates (gender, housing type, employment), but no significant interactions were identified (see Supplemental Table 19) and interaction terms did not improve model fit, χ^2^(5) = 5.87, *p* = .32.

Dog ownership alone (Model 1) did not significantly predict odds of having depression (χ^2^[1] = 2.93, *p* = .09), but when covariates were included in the model (Model 2), dog ownership was associated with higher odds of having depression (OR: 1.65, Model 2 χ^2^[14] = 47.00, *p* <.001, Nagelkerke R^2^ = .07). Model 3 found no significant interactions between dog ownership and race/ethnicity and housing type, but there was a significant interaction between employment status and dog ownership in predicting odds of depression (OR: 0.47, 95% CI [.24, .93], *p* = .03). For individuals who had a dog, those who were employed were less likely (OR: 0.42) to have depression compared to those who were unemployed. However, participants without a dog had similar odds of having depression if they were employed (OR: 0.90) compared to being unemployed. Individuals who reported being unemployed had significantly higher odds of having depression if they owned a dog (OR: 2.15), but for those who were employed, dog ownership was not associated with higher odds of depression (OR: 1.01). Table 4 includes pet, dog, and cat ownership parameters and odds ratios, full regression parameters for all covariates and interaction terms in all models are reported in Supplemental Table 19, 20, and 21.

## Discussion

The purpose of this study was to assess if pet owners in the United States differ systematically on sociodemographic factors. Additionally, we assessed if these patterns were different in dog and cat owners, and if these factors would moderate relationships between HAI and health indicators. Overall, the sample reported similar rates of pet ownership as other estimates in the United States (APPA, 2021). Results did suggest a number of demographic and contextual differences between pet owners and non-pet owners, as well as differences based on dog and cat ownership specifically. For individual-level demographic characteristics, gender was a predictor of pet ownership, with women having higher odds of pet ownership than male participants, but there were no gender differences specific to dog and cat ownership. Similar to past research in the UK (Marsa-Sambola et al., 2016; Westgarth et al., 2010; Westgarth et al., 2012), pet ownership did vary across racial/ethnic identity, with White/non-Hispanic participants having higher odds of pet ownership than other racial/ethnic identity groups, although the specific patterns did differ across dog and cat ownership. In general, HAI research has been plagued by lack of diversity in research participants (Esposito, McCune, Griffin, & Maholmes, 2011; McCune et al., 2014; Mueller, 2021), often with an overrepresentation of White and female participants. Given that there may be systematic differences between pet owners and non-pet owners (and across species) on these demographic characteristics, there is clearly a need for more representative sampling strategies.

Family composition as part of the sociodemographic profile of a household can also contribute to health and well-being. In this sample, pet ownership did not vary based on marital status, but families with children currently living in the home were more likely to own pets (and dogs in particular) compared to families without children. Families may choose to acquire a dog because of the perceived benefits of dog ownership on responsibility and caretaking for children (Fifield & Forsyth, 1999; Paul & Serpell, 1996). However, having children in the home did not predict cat ownership, suggesting that there may be differences in types of animals that are brought into the family based on age and presence of children.

Socioeconomic variables suggested that income alone was not a significant predictor of pet ownership, dog ownership, or cat ownership. Although other research has found relationships between family affluence more broadly and pet ownership (e.g. Marsa-Sambola et al., 2016; Miles et al., 2017), our study found that income alone is not a meaningful predictor of pet ownership. Educational status, which is often used as a proxy for socioeconomic status (e.g. Bornstein, Hahn, Suwalsky, & Haynes, 2003), did differ between pet owners and non-pet owners. Participants with the highest levels of education (bachelor’s degree or higher) had lower odds of pet ownership than those with less than a high school education (with similar patterns for dog and cat ownership), again similar to prior findings in a UK sample (Westgarth et al., 2010). However, other research has shown that higher levels of education were associated with higher likelihood of owning a cat (but lower likelihood of owning a dog; Murray, Browne, Roberts, Whitmarsh, & Gruffydd-Jones, 2010), suggesting that these relationships still warrant further inquiry and may be changing over time. In addition, individuals who were employed had higher odds of pet ownership than those who were unemployed. These findings suggest that the relationship between pet ownership and socioeconomic status is likely not linear, and families in many income brackets own pets. These results point to the importance of understanding the specific needs of families with differing levels of economic resources and employment circumstances.

Features of residential housing may be particularly relevant to pet ownership as it relates to physical space, outdoor space, proximity of people and animals, and the availability of housing that allows pets. Housing type was a consistent predictor of both pet ownership more generally and dog ownership specifically, with multifamily housing associated with lower odds of pet/dog ownership than one-family detached houses. This finding was not replicated for cat ownership, highlighting the potential perceived differences in housing needs for dogs and cats regarding physical space, as well as the more limited availability of dog-friendly housing options, particularly in multifamily housing. The availability of dog-friendly rental housing and the additional costs that may be incurred by having dogs in rental settings is an area of equity in pet ownership that warrants further research. Similarly, individuals from rural areas were more likely to own any type of pet compared to those living in an urban area. This finding further highlights the potential impact of the physical environment as well as pet-friendly housing in different types of residential configurations on decisions to have or acquire pets, and therefore is an important confounder. For example, if health benefits associated with dog ownership are related to walking, access to physical space would be a necessary component of accessing these benefits. Recent research has also identified racial inequities in pet-friendly housing (Rose, McMillian, & Carter, 2020), and therefore the interaction between other sociodemographics and housing type should be assessed in more detail to promote more equitable access to pet ownership.

The second research question assessed the role of these individual and contextual predictors in explaining systematic differences between pet owners and non-pet owners (and by dog and cat ownership) on indicators of health. Pet ownership, dog ownership, and cat ownership were not associated with self-reported overall health or BMI, with or without sociodemographic control variables, although these measures have limitations as robust indicators of health. These findings are not entirely surprising based on the mixed body of literature regarding non-cardiac specific physical health and pet ownership. Both overall health and BMI are highly complex indicators of health that are influenced by myriad individual, contextual, and environmental features of which pet ownership is a small portion.

Within the more specific context of light to moderate physical activity (of the type that would be associated with dog walking), dog ownership did predict higher levels of physical activity, controlling for demographic covariates. However, there were no differences based on pet ownership more generally or cat ownership specifically. These findings are consistent with past research associating physical activity with dogs and dog-walking (e.g. Potter et al., 2021; Westgarth et al., 2019). The demographic predictors associated with both physical activity and dog ownership separately did not interact with dog ownership in predicting activity levels. These findings are not entirely surprising; most dogs require at least minimal outside/walking time every day, which can encourage their owners to engage in light/moderate activity. However, it is important to note that physical activity related to dog ownership may be significantly confounded by breed and/or activity level differences between different types of dogs (Pickup, German, Blackwell, Evans, & Westgarth, 2017) as well as perceptions of breed differences. Individuals who are more active may choose to get more active dogs, and therefore it is a limitation of our analyses that all dog ownership was combined into one category, potentially masking differential relationships.

Pet ownership, dog ownership, and cat ownership were not significantly associated with having long-term physical disability, suggesting that pet owners could have a variety of physical health needs that should be addressed in the context of overall health promotion. Having a pet was associated with higher odds of having a cognitive/learning-related disability, although this appeared to be driven by cat ownership, with no differences between dog owners and non-dog owners on disability status. These findings maintained when controlling for relevant covariates, but there were no interactions between sociodemographics and pet ownership in predicting disability status. It may be that owning a cat presents fewer barriers than owning a dog. Barriers to pet ownership for individuals with a variety of disabilities should be addressed in future research to promote equity in access to pet ownership for all individuals who wish to have companion animals.

Regarding mental health outcomes, pet ownership was associated with higher odds of having an anxiety disorder, and this effect maintained when controlling for covariates. There was a significant interaction between gender and pet ownership where female participants with pets had more than twice the odds of having anxiety compared to female participants without pets. For male participants, the odds of having anxiety were lower for pet owners compared to non-pet owners. These findings raise interesting questions about gender differences in caretaking responsibilities for pets, reasons for acquiring pets, the potential for stress associated with caregiver burden (Spitznagel, Mueller, Fraychak, Hoffman, & Carlson, 2019), and the individual nature of how human-animal relationships may contribute to adaptive coping with stress. Recent research on cat owners has also suggested that owner characteristics such as neuroticism are related in complicated ways to the human-cat relationship (Finka et al., 2019), further underscoring the complexities in who chooses to own what types of pets and how these characteristics interact with mental health. However, the overall number of participants with an anxiety disorder was relatively small and neither dog nor cat ownership were associated with odds of having an anxiety disorder, suggesting the association between pet ownership and anxiety is related to other types of pet ownership. Furthermore, this analysis was significantly limited by the binary representation of gender (there were only five non-cis-gender participants in the sample, perhaps in part due to the wording of the gender question), which did not allow for understanding the unique experiences of non-binary and/or non cis-gender individuals. Therefore, these findings should be interpreted with caution and explored more fully in larger samples with more diversity in gender identity and appropriately worded gender identity questions.

Consistent with some other past research (e.g. Mueller et al., 2018; Sharpley et al., 2020), pet ownership was associated with doubled odds of having depression, and this effect strengthened when controlling for demographic/contextual variables, with similar patterns for both dog and cat ownership. There was also a significant interaction between dog ownership and employment status. For participants who were unemployed, having a dog was associated with twice the odds of having depression compared to those with no dog. For participants who were employed, dog ownership was not associated with any differences in odds of having depression. These findings could have a number of explanations; for example, it may be that individuals who have depression and who are unemployed seek out dog ownership as a way of providing needed social support. Alternatively, perhaps the strain of caring for dog when unemployed exacerbated feelings of depression. These findings also suggest that individuals who are unemployed and are experiencing depression may benefit from specific interventions, perhaps ensuring that they have adequate support to care for their dog while seeking employment, or helping them leverage their relationship with their dog to support improved mental health. Although the nature of this dataset does not allow for exploring any causal relationships, these findings are a clear example of how sociodemographic characteristics, pet ownership, and health outcomes may interact in complex and meaningful ways that should be explored with nuanced methodological approaches.

### Limitations

Although these analyses benefited from the use of a nationally representative dataset, there were still several data-related limitations in addition to those already mentioned (e.g. gender identity variable). While the sample was representative, there may be substantive differences between individuals who take part in panel surveys from the general population. Oversampling was used with the aim of increasing the diversity of the sample, but the participants were still majority White/non-Hispanic (63% of the sample). We also used race/ethnicity as a combined variable due to collinearity and sample size issues but recognize that racial and ethnic identities are distinct and variable, and future research should explore these identities in more detail with larger samples. While this sample was representative and weighted to match population characteristics, the sample size was relatively small and therefore there were correspondingly small numbers of participants who had physical and cognitive disabilities, anxiety disorder, and depression. Similarly, variables such as employment status had subcategories (e.g. retired vs. unemployed and looking for work) that were too small to analyze separately but may be qualitatively different with regard to income and mental health correlates, as well as available time to care for and enjoy a pet. The health indicators used in these analyses were intended to provide an exploratory assessment of overall health, but there are limitations to self-reported health status as a nuanced method of indexing health, which is multi-dimensional and dynamic. Finally, the effect sizes were generally small and the models explained a relatively small percentage of the variance in outcomes. While this is expected, due to the myriad factors that can contribute to health outcomes, these findings should still be interpreted with caution.

The percentage of families with children was also relatively small (25%) compared to the United States at large (40%; US Census, 2020). This study only assessed the experiences of adults, although past research in the UK has shown sociodemographic differences in pet ownership in children and adolescents (Miles et al., 2017; Westgarth et al., 2010). Finally, we only assessed current pet ownership and were not able to explore duration of pet relationships, history of pet ownership, or timing and reasons for acquisition of a pet, which can also be related to demographic variables (Vink, Dijkstra, & Epstude, 2019). These relationships should be explored with robust qualitative and mixed methods research. Furthermore, the conclusions that can be drawn from these analyses should not be over-interpreted, but instead used as a suggestion for what variables and relationships may be useful to explore in future larger-scale studies.

## Conclusions

Overall, the results from these analyses supported the hypothesis that pet owners in the United States are systematically different from non-pet owners on a number of important demographic and contextual indices. Family composition, employment status, and housing type and location in particular seem to be consistently important to explore in the context of pet ownership. It is also clear that research should not combine all pet ownership into one category, and that there are different patterns of pet ownership and health indicators across species. Furthermore, there were differences between pet owners and non-pet owners (as well as between dog and cat ownership) on indicators of health outcomes, even when controlling for covariates. While there were some positive associations between dog ownership and physical health (i.e. physical activity), pet ownership was associated with higher odds of anxiety and depression, with gender and employment status emerging as relevant moderating variables.

The design of this study allowed us to assess dimensions of pet ownership that may be related to systematic inequities, particularly with regard to access to human and veterinary healthcare and barriers to pet ownership. As previously noted, HAI research has long suffered from notoriously non-diverse samples, and the results from this analysis underscore the need for prioritizing more representative sampling approaches, and perhaps over-sampling for certain sociodemographic characteristics. Pet ownership is not a ubiquitous phenomenon; pet owners are systematically different from non-pet owners in ways that are complex, diverse, context-specific, and relevant to indicators of health. Pet ownership is also a dynamic phenomenon, with individuals choosing to have pets in different stages of their lives, perhaps reflective of personal and contextual circumstances. In order to fully understand the potential for pets to impact health and quality of life, future research should design measurement and analysis approaches that are sensitive to the complex systems in which people and pets interact. Research that is sensitive to these important features of the context will yield findings that are more ecologically valid and identify more specifically for whom pet ownership is beneficial and what barriers might exist to positive human-animal relationships.

## Data Availability

The design and secondary data analysis plan for this study were preregistered on the Open Science Framework, and all analysis code is publicly available: https://osf.io/3ctdk/?view_only=cb0ea06867c348ad9f7facf92e93d098. We report deviations from our preregistered protocol in the Supplementary Materials. Due to the proprietary nature of the Ipsos data set and the nature of some data sources, the data set is not currently stored on a public repository and requires IRB approval for access. The dataset is available through protected access from the Tufts University research team. More information on the dataset is available at: https://equityresearch.tufts.edu/the-data/
